# Predictors of Study Attrition in a Randomized Controlled Trial Evaluating a Perinatal Home-Visiting Program with Mothers with Psychosocial Vulnerabilities

**DOI:** 10.1371/journal.pone.0142495

**Published:** 2015-11-10

**Authors:** Stéphanie Foulon, Tim Greacen, Blandine Pasquet, Romain Dugravier, Thomas Saïas, Nicole Guedeney, Antoine Guedeney, Florence Tubach, CAPEDP Study Group

**Affiliations:** 1 Département d’Epidémiologie et Recherche Clinique, AP-HP Hôpital Bichat, Paris, France; 2 Laboratoire de recherche, Etablissement Public de Santé Maison Blanche, Paris, France; 3 Département d’Epidémiologie et Recherche Clinique, CIC-EC 1425, AP-HP Hôpital Bichat, Paris, France; 4 Service de Pédopsychiatrie, AP-HP Hôpital Bichat, Paris, France; 5 U669 PSIGIAM, Institut National de la Santé et de la Recherche Médicale, Paris, France; 6 Institut National de Prévention et d’Education pour la Santé, Saint-Denis, France; 7 Département de Psychologie, Université du Québec à Montréal, Montréal, Canada; 8 Institut Mutualiste Montsouris, Paris, France; 9 INSERM, ECEVE, UMR 1123, CIC-EC 11425, Paris, France; 10 Université Paris Diderot, ECEVE, UMR 1123, Sorbonne Paris Cité, Paris, France; University of Rennes-1, FRANCE

## Abstract

**Objective:**

Randomised controlled trials evaluating perinatal home-visiting programs are frequently confronted with the problem of high attrition rates. The aim of the present study is to identify predictors of study attrition in a trial evaluating a perinatal home-visiting program in France.

**Materials and Methods:**

CAPEDP is a French randomized trial comparing a perinatal home-visiting program using psychologists versus usual care (N = 440). The first assessment was at inclusion into the trial at the 27^th^ week of pregnancy and the final assessment when the child reached the age of two. Attrition rates were calculated at 3 and 24 months postpartum. Stepwise logistic regression was used to identify predictors of *early* (between inclusion and 3 months postpartum) and *later* (between 3 and 24 months postpartum) attrition among social, psychological and parenting factors.

**Results:**

Attrition rates were 17% and 63% at 3 and 24 months respectively. At 24 months, there was significantly more attrition in the control arm (70.6%) compared to the intervention arm (55.2%). Five independent predictors of early attrition were identified: having already had an abortion; having greater attachment insecurity as measured with the Vulnerable Attachment Style Questionnaire (VASQ); having lower global severity of psychiatric symptoms as assessed with the Symptom Check-List (SCL-90) at inclusion, being neither currently employed nor studying; and declaring no tobacco consumption during pregnancy. Being randomized into the control arm, having undergone early parental loss before age 11 and having lower global severity of psychiatric symptoms (SCL-90) at 3 months postpartum were the only variables associated with later attrition.

**Conclusion:**

This study provides key information for identifying mothers who may require specific support to avoid study attrition in trials evaluating a home-visiting program.

## Introduction

A randomized controlled trial (RCT) is widely regarded as the gold standard for assessing the efficacy of medical and behavioral interventions. However, retaining participants in RCTs can be a challenge, with significant risks in terms of being able to generalize from the findings, loss of power with diminishing numbers of participants, or bias associated with selective attrition of intervention or control group subjects [1]. Extended follow-up, minority and low income populations have been identified as risk factors for attrition in numerous RCTs [2–4]. Studies evaluating perinatal home-visiting programs targeting maternal and child health combine these two risks as they generally (1) target women in psychosocially vulnerable situations and who are therefore seen to be particularly in need of such interventions, and (2) seek to evaluate longer term changes in the mothers’ or the children’s behavior. It is therefore not surprising that reported attrition rates are often high in RCTs evaluating this type of program, ranging from 12–18% for studies with assessments six months after inclusion, 9–41% at month 12, 20–22% at month 18, 5–35% at month 24 and 6–37% at month 36 [4–19], as shown in Table 1. Although the question of retention in home-visiting interventions has been extensively explored [20–26], few studies have specifically addressed the issue of attrition in RCTs evaluating these interventions. In an RCT evaluating a postpartum home-visiting program with socially disadvantaged women in Washington DC, women who dropped out from the study before four months were observed to be older and more educated, assigned less weight to the severity of illnesses that their child might contract, showed less empathy towards their child’s needs and provided a less adequate child-rearing environment [4]. Women who dropped out before completion had a lower mean number of prenatal care visits and a higher mean number of children. In Canada, Tough et al.[18] described study attrition rates in an RCT comparing prenatal home-visiting by paraprofessionals versus either care as usual, or care as usual plus a consultation with a nurse. Independent risk factors for dropping out from the study included: being non-Caucasian, not having completed high school, and having lower social support. The parents of mothers who dropped out were more likely to be separated or divorced. However generalising from these studies to RCT evaluating perinatal home-visiting programs in other contexts is problematic. Both of these trials were comparatively short in terms of follow-up (12 and 8 months respectively), with correspondingly reduced incidence of life events with potential implications for attrition compared to longer term programs, such as changes in the mother's employment, her accommodation needs and place of residence, her psychological condition or her relationship with her child, all of which can have significant implications for continuing to participate in the trial. Furthermore, both studies were evaluating North American urban programs in specific local social contexts: extrapolating to other national or local contexts is problematic. Knowledge of predictors of study attrition in other national or regional contexts and, more importantly, of factors associated with early attrition compared to those associated with later attrition in RCTs evaluating home-visiting programs over longer periods of time is of key importance for helping program designers focus on families at risk of leaving the study and exploring adapted retention strategies.

**Table 1:** Study attrition rates in the intervention (I) arm versus the control (C) arm in RCTs evaluating perinatal home-visiting programs.

Study	Number (N) and characteristics of women included in the trial	Attrition rates (%) at								
		4m	8m	12m	18m	24m	36m	4y	6y	7y
Guteilus,1977	N: 47(I) + 48(C) primiparous, African American, low income women aged 15 to 18 in Washington DC, USA						6–6	11–15	19–46	
Larson, 1980	N: 35(I_A_: home visits beginning prenatally) + 36(I_B_: home visits beginning during postpartum) + 44(C) working-class women aged 18–35 in Montreal, Canada				26(I_A_)-25(I_B_)-16(C)					
Olds, 1986	N:116 (intervention arm with home visits beginning prenatally and continuing up to the child’s 2^nd^ birthday) + 184 (control arm with care as usual, but regrouping two sub-groups: i.e. with or without free transportation to prenatal and well-child visits) primiparous women with at least one vulnerability criteria, being either ≤19 years old or a single parent or of low socioeconomic status, in the Appalachian region of New York State, USA			25–26		35–34				
Hardy, 1989	N: 143(I) + 147(C) low income African American women ≥18 years old, in Baltimore, USA					8–10				
Johnson, 1993	N: 141(I) + 121(C) primiparous women living in a deprived area in Dublin, Ireland			10–13						73–69
Kitzman, 1997	N: 228(I) (pre and post natal home visits) + 515(C) (free transportation with developmental screening) primiparous African American women with at least two of the following vulnerability criteria: unmarried, < 12 years education, unemployed; in Memphis, Tennessee					5–7				
Duggan, 1999	N: 373(I)-270(C) families with new-borns identified as being at risk for child abuse or neglect using the Family Stress Checklist, in Hawaii, USA					17–17				
St Pierre, 1999	N: 2213(I)-2197(C) low income families at 24 sites in the USA							40–35		
Armstrong,2000	N: 90(I)-91(C) families with psychosocial vulnerability in Australia	11–12								
Fergusson, 2005	N: 220(I)-223(C) families with psychosocial vulnerability in New Zealand						16–7			
Barlow, 2007	N: 68(I)-63(C) women with psychosocial vulnerability in the UK			9–12			23–28			
Caldera, 2007	162(I)-163(C) women with psychosocial vulnerability in Alaska					15–14				
Kemp, 2011	N: 111(I)-97(C) women with psychosocial vulnerability, in Sydney, Australia			20–17	22–23	23–29	35–40			
Tough, 2007	N: 577(I)-1438(C) middle income urban families, in Calgary, Canada		17							
Katz, 2001	N: 145(I)-140(C) women ≥18 with low income, in Washington DC, USA	19–34	28–41	39–45						
Sharps, 2013	N: 124(I)-115(C) women reporting abuse in the last 12 months, in the USA			30–30						

Attrition rates are presented at different points in time from the beginning of each study. When this information has been made available in the published articles, attrition rates are presented separately for the intervention (I) arm and the control (C) arm: (I-C).

The current study sets out to address these two key questions, using data from the CAPEDP (*Compétences parentales et Attachement dans la Petite Enfance*: *Diminution des risques liés aux troubles de santé mentale et Promotion de la résilience*—Parental Skills and Attachment in Early Childhood: reduction of risks linked to mental health problems and promotion of resilience) trial, an RCT evaluating a home-visiting program in France and specifically targeting child mental health and its major determinants. Women were recruited during the third trimester of pregnancy and followed up to their child’s second birthday.

The objectives of the current study are to describe study attrition in the CAPEDP trial, to identify predictors of early attrition, before the third month post-partum, and also those associated with becoming lost to follow-up at any later point during the project, and to see whether predictors of study attrition in the French context in an RCT using psychologists as home visitors will be similar to those described in the North American studies mentioned above. It must be highlighted that this paper specifically addresses attrition from the RCT in question and not from the home-visiting program itself.

## Materials and Methods

### Study design and participant population

Participants were recruited in the CAPEDP trial, a prospective, multicenter RCT with two parallel arms, using Prospective Randomized Open Blinded Endpoint (PROBE) methodology, with a 27-month follow-up evaluating the impact of a perinatal home-visiting program conducted by trained psychologists and targeting the major modifiable determinants of infant mental health. The program evaluated three primary outcomes: child mental health at the age of two, as well as two potential mediating variables: maternal postnatal depression at three months postpartum and the quality of the home environment when the child was 12 months old.

All consecutive women consulting in the second trimester of pregnancy in nine public maternity wards in the central Paris area and inner suburbs were assessed for eligibility. Eligible women were less than 26 years old, primiparous, less than 27 weeks pregnant at their first assessment session and presented at least one of the three following vulnerability criteria: 1) having less than twelve years of schooling, 2) intending to raise their child alone 3) being eligible, due to lack of personal income, for state-funded health care free of charge. Exclusion criteria were: women who would be impossible to follow up (such as travelers, homeless, or temporary refugees), women receiving social or medical care for other reasons (such as substance abuse, serious mental illness, or other chronic diseases requiring close follow-up), and women who did not consent to participate. A total of 440 women were recruited between December 2006 and March 2009. After completing baseline screening and informed consent procedures at a prenatal medical appointment in one of nine maternity wards in the Paris area, participants were then randomly assigned in a 1:1 ratio to either the CAPEDP intervention or the usual care group and an appointment was made for the first assessment interview at the participant’s home. The protocol is described in detail elsewhere [27], as are the principle results [28,29]. The trial was registered as ClinicalTrials.gov number NCT0039284 and approved by the Institutional Review Board ‘Comité de Protection des Personnes Ile de France IV’ (IRB authorization 2006/37) and by the French Data Protection Authority. Written consent was obtained from all participants.

The CAPEDP home-visiting intervention was tailored to improve mothers’ knowledge and skills with regard to parenting, develop infant-mother attachment security, and enhance social and professional integration. A major specificity of the CAPEDP trial was that the entire home-visiting program was conducted and evaluated by qualified psychologists. It was hypothesized that professionals who were more highly trained in psychology would be more competent in recognizing the elements in play with regard to the determinants of infant mental health, more skilled in acting upon these determinants, and more skilled in evaluating outcomes. The program was designed for psychologists to visit families six times during the antenatal period, eight times in the first three months of the child’s life, 15 times when the child was between 4 and 12 months of age and another 14 times during the child’s second year of life. To optimise adherence to the intervention, families were reminded of upcoming visits by phone or with text messages. Missed home visits were rescheduled within the following week. Home visitors were also encouraged to maintain telephone contact with families between visits. Families that regularly missed home visits or did not respond to phone calls continued to receive regular calls at least once a fortnight from their home visitor; these calls continued through to the end of their planned participation in the study. Finally, letters were regularly sent to each family who had not been in direct contact with their home visitor for a period of over three months without giving any news.

Usual care involved access to Mother and Child Protection Services (*Protection Maternelle et Infantile*: *PMI*) and the community mental health network with no out-of-pocket payment, free antenatal maternity screenings, and a variety of social benefits.

### Assessment procedures

Assessment visits were conducted in both arms during specific home visits by a team of four trained and supervised psychologists, working independently from the psychologists performing the CAPEDP intervention and blinded to group allocation (intervention group or usual care group). Although all evaluators had at least a master’s degree in psychology, they were instructed to provide no support to the mothers except if they observed significant risk situations, in which case they were to immediately inform the principal investigator. Seven home-based assessment visits were scheduled for each family, beginning at the 27th week of pregnancy and then when their child was 3, 6, 12, 18 and 24 months old. All participants were contacted by the evaluation team at every assessment point. In cases where families accepted phone contact but were reticent to receive further home visits, assessments took place over the telephone, excluding those instruments that required direct observation. If phone contact proved to be impossible, questionnaires were sent to families via the post. An assessment visit typically lasted approximately 90 minutes. The mothers received no payment for their participation. Socio-demographic data collected included: mother’s age; country of birth; number of years in France if migrant; number of years in current home; educational level; employment status; income; perception of financial situation; access to health services; use of social services; tobacco, alcohol and drug consumption. Data related to family structure included marital status, number of years with current partner, and death, separation or divorce of the mother’s own parents. Medico-obstetrical characteristics such as prior voluntary termination(s) of pregnancy, planning of the current pregnancy, and the number of health visits in the preceding year related to the current pregnancy, including visits to gynaecologists, midwives, paediatric nurses, and PMI services, were collected. All these data were collected at the initial assessment interview during the prenatal period, with the exception of the mother’s employment status which was assessed at both the first and the second assessment interviews. Data related to parenting were collected using validated scales investigating the mother’s knowledge of infant development (Knowledge of Infant Development Inventory, KIDI), her perception of her attitude, competence and emotional investment towards her child using the Parental Cognitions and Conduct Toward the Infant Scale (PACOTIS) [30], the quality of the home environment using the Home Observation for the Measurement of the Environment (HOME) inventory [31,32] and the Parental Stress Inventory (PSI) [33]. Maternal psychological characteristics were assessed with the Edinburgh Postnatal Depression Scale (EPDS) [34,35], a scale measuring pre- and post-natal depressive symptoms, the Symptom CheckList 90 (SCL-90) [36] with ten subscales investigating psychological distress, and the Vulnerable Attachment Style Questionnaire (VASQ) [37] measuring two domains of the mother’s attachment styles. Table 2 presents the assessment schedule for the instruments used.

**Table 2:** Assessment Schedule.

Instrument	Assessment	Prenatal	3 months after birth
Knowledge of Infant Development Inventory (KIDI)	Mother’s knowledge of infant development	X	X
Parental Cognitions and Conduct Toward the Infant Scale (PACOTIS)	Parenting: mother’s perception of her attitude and behaviour towards her child, of her competence and of her emotional investment with the child		x
Home observation for the Measurement of the Environment (HOME)	Quality of the home environment (quality and quantity of stimulation and support available to the child in the home environment)		x
Parental Stress Inventory (PSI)	Parental Stress		x
Edinburgh Post-partum Depression Scale (EPDS)	Pre and postpartum depression	X	x
Symptom Check-list (SCL90)	Psychological disorders in the mother	X	x
Vulnerable Attachment Style Questionnaire (VASQ)	Mother’s attachment style	X	

### Definition of attrition

The present study examines attrition from the trial, and therefore includes participants from both the intervention group and the control group. Women who underwent medical termination of pregnancy, women whose babies died, women wrongly included in the CAPEDP study and women whose consent forms got lost were not considered as having dropped out. The initial attrition of women who dropped out immediately after randomization but before the first assessment interview (T1) at their 27^th^ week of pregnancy can only be described but not explained because no data, apart from eligibility criteria, were collected concerning these women. For the purposes of the present study, which examines factors associated with attrition in the CAPEDP assessment protocol, *early attrition* is defined as having taken part in the first assessment interview (T1), in the prenatal period, but in no further assessment interviews. *Later attrition* concerns mothers who were in the study at the time of the second assessment interview (T2), which took place when their child was three months old, but who then interrupted their participation in assessment interviews at some later point before the final assessment due to be conducted when their child reached the age of two. We chose to study predictors of *early attrition* separately from those of *later attrition* in order to be able to identify the specific characteristics of women whom the research team proved unable to engage with at any point beyond the initial assessment interview when the women had not yet become mothers. Indeed, numerous factors enter into play from a theoretical point of view. A key issue in France is the fact that, for mothers who have a job, state-funded maternity leave ends three months after the child is born. Going back to work could influence their availability and thus attrition rates in the study. Missing one or more of the interim assessment interviews was not considered to be attrition as long as the mother took part in the final assessment. Two women from the intervention group who missed the initial assessment interview during pregnancy, but who then rejoined the assessment procedure at some later point have been included in the present analysis.

### Statistical analysis

Predictors of early and later attrition were analyzed separately. We first examined univariate relationships between potential predictors and early or later attrition. The variables tested were those reported as being significant in previous studies: age, immigration status, educational level, low income, social support, tobacco and alcohol consumption, marital status, mother's own parents being separated, numbers of prenatal visits and quality of the home environment [4,18] and those related to retention and attrition from a theoretical clinical point of view with regard to social and microsocial stability (recent immigration, unemployment, attachment insecurity). It was also hypothesized that women who felt the need for psychological support or guidance on parenting would be more likely to be retained in the present study, because both the intervention and the assessment home visits were conducted by trained psychologists. Descriptive statistics (frequencies, percentages, means, standard deviations) were used to describe population characteristics. The χ^2^ test (or Fisher test) and student test (or Wilcoxon test) were performed for inter-group comparisons, as appropriate. All tests were two-sided, and significance was accepted at the 5% level (α = 0.05). Multivariate logistic models were used to explain early or later attrition, with a stepwise selection procedure based on backward elimination based on p-values (α_drop_ = 0.10). The initial set of explanatory variables included in the multivariate models was determined using two sources: 1/ variables associated with early or later attrition with a p-value < 0.20 in univariate analysis; 2/ variables identified in the scientific literature as being associated with attrition in RCTs evaluating perinatal home-visiting programs, but not significant in univariate analysis in the present study. Missing data were handled using multiple imputation, when needed (later attrition analysis). Analyses were performed using SAS version 9.2 and R version 2.15.2.

## Results

**Fig 1:**
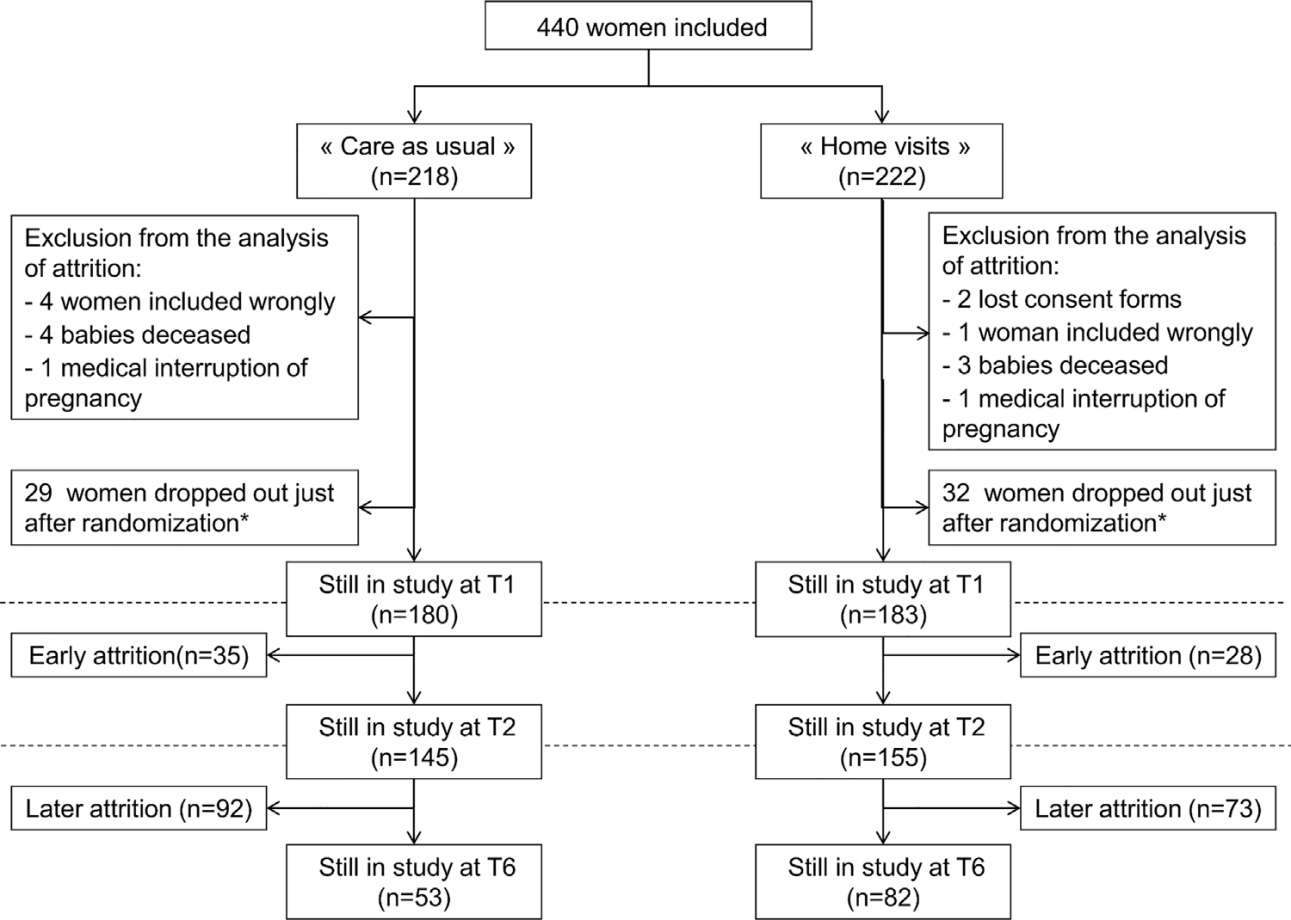
Attrition from assessment procedures after the first prenatal assessment (T1, at the 27^th^ week of pregnancy), when the child was 3 months old (T2) and at the child’s second birthday (T6, final assessment). * No data were collected for women who dropped out between randomization and T1 apart from eligibility criteria.

As described in Fig 1, among the 440 women included in the CAPEDP trial, nine were excluded from the current analysis, due to medical termination of pregnancy or the death of their baby and seven because they were wrongly included or their consent form was lost. Between randomization and the first assessment interview (T1) at their 27^th^ week of pregnancy, 61 women (14.4%) dropped out. No association was observed between this type of attrition and the trial arm into which these women had been randomized (p = 0.76).

### Description of attrition

The present analysis was conducted for the 363 women still in the study for their first assessment interview during their third trimester of pregnancy (*early attrition analysis*) and the 300 women still in the study at the time of the second assessment interview, when their child was three months old (*later attrition analysis*). It is to be noted that two women in the intervention group who could not be contacted for the T1 interview then proceeded to participate in one and three later assessment interviews respectively. A further seven mothers from the intervention group continued receiving the home-visiting intervention after their child was three months old but refused the second and all subsequent assessment interviews; two of these mothers continued on in this way well into the second year of the intervention.

At inclusion, the 363 women were generally young (mean (SD) age = 22.3 (2.4)), with low educational levels (74% had no higher education) and low income (46.2%, due to lack of personal income, were eligible for government-funded health care with no out-of-pocket payment). Just over half (51.8%) were born abroad. Of these 363 women who participated in the first assessment interview at their homes at T1, 63 (17.4%) participated in no further assessment interviews in the CAPEDP study (19.4% in the control group versus 15.3% in the intervention group; p = 0.30). A further 165 women ceased participation in assessment interviews at some later point during the study. In all, 228 women (63%) who were assessed at T1 proved not to be available for the final CAPEDP assessment interview when their child reached the age of two. The attrition rate for the final assessment interview was significantly greater in the control group (70.6%) than in the intervention group (55.2%) (p = 0.002).

### Predictors of early attrition

In univariate analysis (Table 3), women in the early attrition group were less likely to be employed or studying at inclusion (22.2% versus 40.1%, p = 0.008) and were more likely to have had at least one abortion (37.7% versus 24,7%, p = 0.038). In multivariate analysis (Table 4), two variables identified in the scientific literature as being associated with study attrition in RCTs evaluating perinatal home-visiting programs, but that did not prove to be significant in univariate analysis (p < 0.20) in the present study, were forced into the model: maternal educational level (having less than 12 years of schooling) and country of birth (being born elsewhere than in France). Multivariate analysis revealed that currently being employed or studying, or never having undergone an abortion halved the likelihood of attrition. Having a higher overall level of psychological distress (Global Severity Index of the SCL-90) at T1 and smoking during pregnancy were also associated with lower attrition. In contrast, women who had higher levels of insecurity in the VASQ attachment scale at T1 were more likely not to have participated in the following assessment interviews.

**Table 3:** Variables associated with early and later attrition (univariate analysis).

		Early attrition (N = 363)			Later attrition (N = 300)		
		No early attrition (N = 300)	Early attrition(N = 63)	p-value	No later attrition (N = 135)	Later attrition(N = 165)	p-value
**Mother’s socio-demographic characteristics**							
Age (years)		22.3 (2.4)	22.2 (2.4)	0.594	22.5 (2.4)	22.2 (2.4)	0.370
Country of birth				0.650			0.276
	France	142 (47.7)	32 (50.8)		69 (51.1)	73 (44.8)	
	Other country	156 (52.3)	31 (49.2)		66 (48.9)	90 (55.2)	
Recent immigrant (< 5 years)				0.920			0.787
	Yes	55 (18.6)	11(18.0)		24 (17.9)	31 (19.1)	
	No	241 (81.4)	50 (82.0)		110 (82.1)	131 (80.9)	
Number of years in current home		2.9 (4.7)	1.9 (3.5)	0.093	3.2 (4.8)	2.6 (4.7)	0.041
Educational level				0.807			0.079
	Less than 12 years	222 (74.5)	46 (73.0)		94 (69.6)	128 (78.5)	
	12 years or more	76 (25.5)	17 (27.0)		41 (30.4)	35 (21.5)	
Currently employed or studying^a^				0.008			0.470
	Yes	119 (40.1)	14 (22.2)		36 (28.1)	46 (32.2)	
	Non	178 (59.9)	49 (77.8)		92 (71.9)	97 (67.8)	
Perceived financial situation				0.729			0.178
	Comfortable	179 (64.9)	39 (67.2)		89 (69.0)	90 (61.2)	
	Poor / Very poor	97 (35.1)	19 (32.8)		40 (31.0)	57 (38.8)	
Low income (CMU / AME)				0.120			0.192
	Yes	133 (44.8)	35 (55.6)		54 (40.6)	79 (48.2)	
	Non	164 (55.2)	28 (44.4)		79 (59.4)	85 (51.8)	
Has consulted a social worker in the year preceding inclusion				0.456			0.346
	Yes	171 (57.8)	39 (62.9)		74 (54.8)	97 (60.2)	
	No	125 (42.2)	23 (37.1)		61 (45.2)	64 (39.8)	
Tobacco consumption during pregnancy				0.101			0.801
	Yes	66 (22.1)	8 (12.9)		29 (21.5)	37 (22.7)	
	No	232 (77.9)	54 (87.1)		106 (78.5)	126 (77.3)	
Alcohol consumption during pregnancy				ND^b^			0.262
	Yes	27 (9.1)	2 (3.2)		15 (11.1)	12 (7.4)	
	No	271 (90.9)	60 (96.8)		120 (88.9)	151 (92.6)	
**Family situation**							
Marital status				0.787			0.297
	Married/common law	169 (56.7)	34 (54.8)		81 (60)	88 (54)	
	Single	129 (43.3)	28 (45.2)		54 (40.0)	75 (46)	
Intends to raise her child alone				0.461			0.434
	Yes	78 (26.1)	19 (30.6)		32 (23.9)	46 (27.9)	
	No	221 (73.9)	43 (69.4)		102 (76.1)	119 (72.1)	
Years living with partner		1.1 (1.5)	1.1 (1.5)	0.920	1.1 (1.5)	1.0 (1.4)	0.808
Death of a parent or separation of mother’s parents before age 11				0.881			0.020
	Yes	117 (43.3)	24 (44.4)		43 (35.5)	74 (49.7)	
	No	153 (56.7)	30 (55.6)		78 (64.5)	75 (50.3)	
**Medico-obstetrical characteristics**							
Has had at least one abortion				0.038			0.483
	Yes	73 (24.7)	23 (37.7)		36 (26.7)	37 (23.1)	
	No	222 (75.3)	38 (62.3)		99 (73.3)	123 (76.9)	
Number of health visits related to pregnancy		8.3 (5.1)	9.1 (6.2)	0.257	8.9 (4.9)	7.8 (5.2)	0.074
**Parenting**							
KIDI score^a^		17.8 (8.8)	19.0 (9.4)	0.337	22.9 (7.5)	21.4 (9.3)	0.153
PACOTIS at T2				-			
	Parental coercion	-	-		1.7 (1.5)	1.4 (1.5)	0.057
	Parental competence	-	-		1.3 (1.2)	1.2 (1.0)	0.215
	Impact perception	-	-		2.4 (1.8)	2.5 (2.1)	0.606
HOME score at T2				-			
	Parental responsivity	-	-		8.3 (1.7)	8.0 (1.7)	0.062
	Acceptance of the child	-	-		5.0 (0.9)	5.0 (1.0)	0.259
	Organization of the environment	-	-		4.9 (1.0)	4.8 (1.0)	0.392
	Learning materials				3.5 (1.4)	3.2 (1.4)	0.059
	Parental involvement	-	-		3.0 (1.0)	2.9 (1.0)	0.617
	Variety in experience	-	-		2.0 (0.9)	2.0 (0.9)	0.422
PSI global score at T2		-	-		53.9 (11.1)	55.4 (10.7)	0.242
	Parental stress	-	-		31.4 (6.9)	31.8 (7.0)	0.669
	Dysfunctional interaction	-	-		22.4 (5.6)	23.6 (5.9)	0.090
**Maternal psychological characteristics**							
Depression (EPDS global score)^a^		10.8 (5.8)	10.6 (4.9)	0.830	9.1 (5.3)	8.4 (5.7)	0.288
Psychiatric symptoms (SCL-90 global score)^a^		0.8 (0.5)	0.7 (0.4)	0.147	0.6 (0.4)	0.5 (0.4)	0.125
Attachment security (VASQ global score at T1)		66.2 (8.2)	67.4 (7.4)	0.299	66.6 (8.2)	65.9 (8.1)	0.429
	Insecurity subscale	33.6 (6.2)	34.9 (5.6)	0.129	33.4 (6.5)	33.8 (6.0)	0.630
	Proximity-seeking subscale	32.6 (5.2)	32.5 (5.0)	0.851	33.2 (5.0)	32.1 (5.3)	0.069
**Trial-related factors**							
Trial arm				0.297			0.004
	Home-visits	155 (51.7)	28 (44.4)		82 (60.7)	73 (44.2)	
	Care as usual	145 (48.3)	35 (55.6)		53 (39.3)	92 (55.8)	
Recruitment center (9 centers, not listed here)				0.772			0.222

Data presented as N (%) or mean (SD).

^a^ variable collected at T1 for early attrition and at T2 for later attrition.

^b^ ND = test not computed due to low numbers

**Table 4:** Predictors of early and later attrition using multivariate analysis adjusted for country of birth (France vs elsewhere) and educational level (<12 vrs vs ≥ 12yrs).

	Early attrition			Later attrition		
	Adjusted OR	95% CI	p-value	Adjusted OR	95% CI	p-value
Number of years spent in current home	0.93	(0.86–1.00)	0.092	-	-	-
Maternal currently employed / studying (Yes vs No)^a^	0.42	(0.21–0.80)	0.011	-	-	-
Tobacco consumption during pregnancy (Yes vs No)	0.41	(0.16–0.94)	0.047	-	-	-
Death of a parent or separation of mother's parents before age 11 (Yes vs No)	-	-	-	2.00	(1.20–3.33)	0.008
Has had at least one abortion (Yes vs No)	2.20	(1.17–4.11)	0.014	-	-	-
Number of health visits related to the pregnancy	-	-	-	0.96	(0.91–1.01)	0.100
Parent-child dysfunctional interaction (PSI) at T2	-	-	-	1.04	(1.00–1.09)	0.070
Psychiatric symptoms (SCL-90 global score)^a^	0.37	(0.16–0.79)	0.013	0.42	(0.19–0.93)	0.033
Attachment (VASQ insecurity subscale at T1)	1.06	(1.01–1.12)	0.026	-	-	-
Trial arm (Usual care vs Home-visits)	-	-	-	1.92	(1.19–3.11)	0.008

^a^ Variable collected at T1 for early attrition and at T2 for later attrition.

### Predictors of later attrition

Predictors of later attrition were somewhat different from predictors of early attrition. In univariate analysis (Table 3), women who completed the final assessment interview were more likely to have been randomized into the intervention group (60.7% versus 44.2%, p = 0.004) and to have been living for a longer period of time in their current homes (3.2 versus 2.6 years, p = 0.041). They were also less likely to have experienced early parental loss or separation (35.5% versus 49.7%, p = 0.020). In multivariate analysis, also adjusted for country of birth and educational level, three variables predicted later attrition: being in the control group, the mother having undergone early parental loss or separation (death or separation of the woman’s own parents before she was 11 years old), and the mother presenting a higher overall level of psychological distress (Global Severity Index of the SCL-90) at T2. Being in the “care as usual” group almost doubled the risk of dropping out.

## Discussion

In the CAPEDP trial, a randomized trial evaluating the impact on child mental health and its major determinants of a home-visiting program conducted by psychologists during the perinatal period, rates of attrition were 17% and 63% when the children were three months old and 24 months old respectively. Attrition rates differed between the two arms of the trial, with more attrition in the control group (care as usual) by the time the children reached 24 months of age than in the intervention group (home-visiting program). Predictors of early attrition (before the child was 3 months old) included having had an abortion and having higher attachment insecurity as measured by the VASQ attachment scale. Being employed or currently studying or doing training, tobacco consumption during pregnancy and presence of psychiatric symptoms were positively associated with early retention. For women who initially adhered to the trial and who were still in the study when their child was three months old, three variables predicted later attrition: being randomized into the control arm, the mother having undergone early parental loss, and the mother presenting lower global severity of psychiatric symptoms (SCL-90) at 3 months postpartum.

The study attrition rate in the CAPEDP trial at the time the children were three months old (i.e. six months after the beginning of the trial) is similar to that observed in other randomized trials evaluating home-visiting programs [4–16,38]. However, it is markedly higher at the end of the program at the child’s second birthday, compared to other trials. This discrepancy may be explained by the fact that the majority of these other studies took place in the USA, whereas in the highly generous French context, care as usual includes easy access for all to perinatal healthcare and child and adult community mental health services with no out-of-the pocket payment. Furthermore, the fact that the CAPEDP program provided no financial or material incentive to participants may well have influenced attrition significantly. Indeed, incentives can play a major role with regard to participant retention both in RCTs in general [39] and in parenting programs in particular [40].

The difference in attrition rates in the control group compared to the intervention group increased throughout the present study. Clearly, participants who appreciated the home-visiting program may have been more likely to remain in the trial and participate in assessment interviews. However, from a more practical point of view, women in the intervention group may simply have been more easily locatable for the assessment team, given their more frequent contacts with the program. Katz et al. [4] observed a similar trend, with higher attrition in the control group at 4 and 8 months. However, this difference disappeared by the final interview at 12 months, in contrast to the CAPEDP study where the attrition rates remained significantly higher in the care as usual group, a phenomenon possibly related to easier overall access to support in the generous French health and social care system. In the present study, it was also found that women with higher psychiatric symptom scores (SCL-90) were more likely to remain in the trial’s assessment protocol. Although the Hawthorne effect cannot be excluded, this phenomenon may well be related to one of the major specificities of the CAPEDP study: all members of both the home-visiting and the assessment teams were qualified clinical psychologists. Providing professional skills corresponding to the needs of participants in this perinatal mental health promotion intervention, with all assessment home visits being conducted by professional psychologists, may well have increased the likelihood that the mothers in question would continue to accept the assessment home visits.

The higher VASQ insecurity scores in subjects who prematurely abandoned the study can be understood in the light of attachment theory. Howe [41], Mikulincer & Shaver [42] and Mallinckrodt et al.[43] underline the possible impact of individuals’ internal working models on their participation in both treatment and research interventions. Attachment insecurity is associated with having an idea of oneself as not being worthy of other people’s interest, and of not being able to count on other people, who are felt to be unreliable or even ill-meaning. Insecure subjects undervalue the impact they might have on other people or on a given situation, particularly when under stress. They may therefore be more prone to dropping out, having a negative perception of the assessment team, or seeing themselves and their participation as being unimportant, as having no possible effect on the research process. The insecure subject also finds it hard to believe that the support the program claims it is going to provide will be of any real help. A meta-analysis by Diener et al. [44] on attachment security and working alliance gives indirect support to this theoretical model, by showing that individuals with secure attachment patterns are likely to develop stronger working alliances with their therapist across different treatment settings. Similarly, the impact of attachment style on home visiting outcomes has been amply demonstrated [45,46].

The fact that having a higher initial attachment insecurity score is not associated with later attrition can be understood in the light of research on working alliances: in relationships with professionals that have been maintained over a certain time, the quality of the relationship itself becomes a determining retention factor, counteracting the negative impact of the individual’s insecure attachment profile. However, it must be underlined that, although the present theoretical analysis is clearly tempting, using attachment security as a primary explanation for attrition in the present study is forcedly speculative. Further research is needed specifically targeting the potentially complex interactions involved.

The following limitations should be considered when interpreting the present results. Firstly, no data could be collected concerning the 61 women who dropped out just after randomization, apart from eligibility criteria. This initial attrition may have been linked to the fact that the trial recruitment process was conducted at a crucial point in the health care pathway: in the second trimester of pregnancy in the same maternity ward in which they were due to have their babies. Although all eligible subjects were informed, both orally and in writing, that they were free to participate or not in the study and that, if they accepted, they were free to withdraw at any moment with no impact on their access to care or the quality of care provided, some women may have not taken sufficient time to reflect upon the implications of participating or may have wanted to please their maternity doctor by agreeing to participate, although without giving enough thought to what their participation would entail. Secondly, despite the large number of participants initially recruited, the present study may well have lacked sufficient statistical power to identify other potential predictors of attrition, particularly with regard to later attrition. Thirdly, the present study is more exploratory than confirmatory, due to the currently limited number of published findings on study attrition in RCTs evaluating home-visiting programs. The present results need to be confirmed in further studies.

Despite these limitations, this study has a number of major strengths: 1) the use of a large number of validated psychological scales exploring different aspects of the women’s psychological state and their interaction with their child, factors that were potentially associated with study attrition; 2) a broad range of potential predictor variables, including all those identified in other studies having investigated this question; 3) a dynamic approach aiming to identify predictors of later attrition in women who had initially adhered to the assessment procedure, thus revealing vulnerability factors in the postpartum period that may differ from those observed during pregnancy (e.g. maternal employment) or that were not available before the child was actually born (e.g. parenting factors). For example, with regard to maternal employment, having a job was associated with lower rates of early attrition, perhaps a sign of geographical stability or of greater psychosocial integration. However, in the postpartum period, being currently employed was no longer a predictor of retention, arguably because women who returned to work at the end of their maternity leave were less available to participate in the study, thus cancelling out the positive effect observed initially. Taking into account predictors of early and later attrition may be useful for fixing stricter eligibility criteria and not including subjects who will in all likelihood drop out. It may also be useful to adapt retention procedures according to each participant’s personal psychosocial characteristics, paying particular attention to the fact that these characteristics may well vary from one period to the next during the study.

In conclusion, the present study delivers a number of key messages for researchers designing RCTs evaluating perinatal home visiting programs or indeed any intervention addressing psychosocially vulnerable populations. Quite clearly the mechanisms underlying study attrition are more complex than the commonplace idea that the more psychosocially vulnerable subjects are more likely to drop out. Designers of RCTs evaluating programs for populations with psychosocial vulnerabilities need to carefully consider the risk of potentially high attrition rates at the different assessment points throughout their programs. Retention strategies should pay particular attention to subjects in the care as usual group, as they have less frequent overall contact with program teams. With regard to perinatal home visiting programs—and this is a key result from the present study—particular care needs to be taken to address factors associated with attrition at different phases in the perinatal period. With regard to preventing drop-out in the early phase of such programs, specific attention should be paid to addressing factors associated with early attrition, including having had an abortion, being unemployed or not currently studying, or having greater attachment insecurity. A key issue is to organize home visits by professionals whose skills correspond to the specific needs of the families being visited, and this remains true not only for the intervention itself, but also for the assessment procedures. In the current study targeting mental health promotion, a study in which all home visits, including assessment home visits, were conducted by trained psychologists, the presence of psychiatric symptoms in the future mother at inclusion was positively associated with early retention in the assessment protocol. With regard to later attrition, the three predictors observed in the current study were early parental loss for the mother herself (death or separation of the woman’s own parents before she was 11 years old), being randomized into the “care as usual” study arm and having lower severity of psychiatric symptoms. Addressing these questions is of key importance for future research.

Finally, an obvious trap for all such studies–and not only in the area of perinatal home visiting programs—is what could be defined as initial attrition, where eligible subjects accept the invitation to participate in the study, sign their consent forms, but then proceed to disappear immediately after randomization and before the initial assessment interview. Although recruitment procedures in the present study systematically informed potential participants that refusing to participate would have no impact on their access to care, it is particularly important to give potential participants time to reflect on the pros and cons of becoming involved in such long term studies and to make it quite clear what the actual assessment procedures involve, over and above the intervention itself. This is all the more so in countries like France with such generous and easy-to-access healthcare systems. A further general recommendation, with relevance to clinical trials in all domains involving long-term participation of research subjects: randomization should take place *after* the initial assessment interview, which was not done in the present study for logistical reasons. Similarly, providing incentives for participation in assessment procedures should be given serious consideration.
